# Getting a healthy start: The effectiveness of targeted benefits for improving dietary choices

**DOI:** 10.1016/j.jhealeco.2018.02.009

**Published:** 2018-03

**Authors:** Rachel Griffith, Stephanie von Hinke, Sarah Smith

**Affiliations:** a Institute for Fiscal Studies and University of Manchester, United Kingdom; b University of Bristol and Institute for Fiscal Studies, United Kingdom

**Keywords:** Dietary choices, Targeted benefits, Healthy Start scheme

## Abstract

There is growing policy interest in encouraging better dietary choices. We study a nationally-implemented policy – the UK Healthy Start scheme – that introduced vouchers for fruit, vegetables and milk. We show that the policy has increased spending on fruit and vegetables and has been more effective than an equivalent-value cash benefit. We also show that the policy improved the nutrient composition of households' shopping baskets, with no offsetting changes in spending on other foodstuffs.

## 1 Introduction

Increasing rates of obesity and diet-related disease are major challenges across the developed world, leading to growing interest amongst the policy community in how to improve dietary choices (Lancet, 2011; Gortmaker et al., 2011). One possible way for the government to improve dietary choices among low-income households is to target benefits on the purchase of healthy food, such as fruit and vegetables. This type of policy has been highlighted as part of the UK government's recent Childhood Obesity plan for action (HM Government, 2016). In the US, the Special Supplemental Nutrition Program for Women, Infants and Children (WIC) already provides vouchers to low-income pregnant/post-partum women, infants and children that can only be spent on specific healthy foods (see e.g. National Academies of Sciences, Engineering, and Medicine, 2017), while targeting in the Supplemental Nutrition Assistance Program (SNAP; formerly Food Stamps) has been discussed due to its large number of recipients and their high levels of obesity.

Our contribution in this paper is to study the impact of the UK Healthy Start scheme, a large-scale, nationally-implemented scheme that distributes vouchers that can only be spent on specific healthy foods (fruit, vegetables and milk) to low-income households with young children with the aim of increasing expenditure on fruit and vegetables, ideally feeding through into consumption. Standard economic theory predicts that the effect of such vouchers will be greatest for distorted consumers (i.e. those who would in the absence of the vouchers spend less than the value of the vouchers on the targeted good), and will be equivalent to cash for infra-marginal consumers (i.e. those who would in the absence of the vouchers spend at least the value of the vouchers on the targeted good). It is therefore an empirical question whether spending on fruit and vegetables^1^ increased as a result of the reform, and also whether the vouchers improved the overall nutrient composition of households’ shopping baskets, since recipient households could respond by adjusting their spending on other food items, with possible nutritional consequences.

Our identification strategy exploits the introduction of the Healthy Start scheme and a discontinuity in eligibility, using a difference-in-differences approach and rich panel data. We compare the change in behaviour before and after the scheme was introduced of households that are eligible for the vouchers to the change in behaviour across a group of similar households that are ineligible. Both treatment and control groups consist of low-income households. Eligibility for the vouchers is determined by the age of children: low-income households with children aged 0–3, or where the woman is at least three months pregnant, are eligible. Low-income households with a woman in the period just before being pregnant or with children aged 4–8 act as a control group of not eligible.

On average, eligible households in our sample received an additional £16.90 (approx. $24) in vouchers per month. Our main results indicate that mean monthly fresh fruit and vegetable expenditure of eligible households increased by approximately £2.43 ($3.50) per month, equivalent to a 15 per cent increase in spending compared to pre-reform levels. We also find that the effect of the vouchers is larger than an equivalent-value cash benefit.

Using the same difference-in-differences approach, we also show that the scheme was associated with significant improvements in the overall nutrient composition of households' shopping baskets. We test for effects on key nutrients, defined over all foods in households’ shopping baskets. We find that levels of fibre, beta-carotene (vitamin A), potassium, iron and zinc increased, while levels of sugar and fat did not change. Furthermore, we find a significant increase in the proportion of households meeting their recommended Reference Intakes for iron and potassium, suggesting an overall improvement in the nutritional content of the shopping basket.

Key to our analysis is the rich data we use. We have panel data that include detailed and precise information on households entire shopping basket, including all food brought into the home, reducing concerns about measurement error and allowing us to identify the effect of the reform. The precise nature of the data allows us to cleanly identify expenditure on products that can be purchased with the vouchers. The fact that we observe the entire food basket of the household also allows us to look at the broader effects of the scheme on the nutrient content of foods purchased, as well as at the potential effects of the scheme on purchases of other foodstuffs. Panel data allow us to control for unobserved heterogeneity in the levels of purchases across households and to be able to use information on household spending prior to the introduction of the scheme to distinguish between households that are likely to be distorted and those likely to be infra-marginal.

Our paper is closely related to an existing literature on the effect of targeted benefits (for an overview, see Currie and Gahvari, 2008). Most recently, Hoynes et al. (2016) use variation in the roll out of Food Stamps to show that it had significant effects on long run child outcomes. Examining the initial roll out of the programme, Hoynes and Schanzenbach (2009) find that most households were infra-marginal. Moffitt (1989) and Whitmore (2002) reach a similar conclusion, investigating cash out experiments. Related, Cunha (2014) looks at in-kind food transfers in Mexico for a set of staple food stuffs. Exploiting randomization during the programme’s roll out, he found consumers to be infra-marginal with respect to overall total food consumption, but he found variation with respect to the individual foods that are distributed. Our contribution in this paper is to focus on a scheme that is more specifically targeted on a narrow range of healthy food products.^2^ There are also some similarities between the Healthy Start scheme and the recent US Healthy Incentive Pilots, which trialled a 30% price subsidy for a randomly-selected sub-group of SNAP recipients and found a 25% increase in fruit and vegetables consumption, with survey respondents indicating that they purchased larger amounts and a greater variety of fruit and vegetables (USDA, 2013).

Our paper is also related to recent empirical studies on the effect of information on dietary choices (see for example Bollinger et al., 2010; Capacci and Mazzochi, 2011) and “nudge effects” created by the labelling of benefits (see for example Kooreman, 2000; Abeler and Marklein, 2010; Beatty et al., 2014; Benhassine et al., 2015) which may operate via the kind of mental accounting suggested by Thaler, 1985, Thaler, 1999. Some features of the programme might have been expected to affect behaviour, beyond the direct economic incentive effects. For example, the vouchers could signal the importance of healthy eating, in particular fruit and vegetables, especially since health professionals played an important gatekeeper role; health professionals were also expected to provide advice on healthy eating, as well as administer the program. One could argue that our analyses therefore evaluate this “package” of services, rather than simply the economic incentives. In further analyses, however, we show that the vouchers increased spending only among distorted, not infra-marginal, households. Since behavioural mechanisms would affect all households, while standard economic incentives are stronger for distorted households, these results suggest that these other features did not have an effect in this context, and that the financial incentives provided the main channel through which the benefits worked.

The structure of the rest of the paper is as follows. The next section presents details of the scheme and discusses its likely effect on behaviour. Section 3 describes the data. Section 4 presents our empirical strategy and main empirical results, with further analyses in Section 5. The final section summarises and provides a concluding discussion.

## 2 Healthy Start Vouchers

### 2.1 The scheme

The Healthy Start scheme was rolled out nationally across the UK on 27 November 2006, and the government reconfirmed its commitment to the scheme in the Childhood Obesity plan for action (HM Government, 2016).^3^ It provides vouchers to low-income pregnant women and low-income households with children up to and including age three, which they can spend on plain (i.e. no added ingredients, such as sugar or seasoning) fresh fruit and vegetables, cow’s milk or infant formula^4^. Low-income is defined as receiving means-tested benefits. Eligible households receive weekly vouchers for each eligible household member: one for a pregnant woman and for children aged 1, 2 or 3, and two vouchers for each child in their first year. The monetary value of a Healthy Start Voucher was initially set at £2.80 ($4.70) per voucher per week, and increased to £3.00 ($5.05) in April 2008.^5^ In our sample, eligible households on average received £16.90 in vouchers per month.

Health professionals play a key gatekeeper role in the scheme. In the UK, there is a comprehensive state-provided system of maternity and early years’ healthcare. Every pregnant woman is allocated to a midwife unit in a Children’s Centre, a GP surgery or hospital and will have up to ten antenatal appointments for her first child and seven appointments for subsequent children. Care continues after birth through the Healthy Child Programme, a series of regular reviews, screening tests and vaccinations led by a health visitor. The Healthy Start scheme is administered by midwives and health visitors, and these health professionals were given a pivotal role in introducing households to the scheme and countersigning the application form. The fact that health professionals had to indicate on the form that they had given the appropriate advice on healthy eating indicates the importance given to the additional features of the scheme. Take-up of Healthy Start Vouchers is high, in part due to the way it was introduced through the existing healthcare system: according to official figures, 79–80% of all eligible households receive the vouchers, and 90% of the vouchers that are issued are used. Government research shows limited abuse of the scheme, with official figures indicating that only 12 out of every one million vouchers being used for purposes other than those for which they were intended, including re-sale (Department of Health, 2009). To the extent that the role of health professionals in the scheme affected fruit and vegetable spending, in addition to any financial incentives discussed below, our estimated effects include this.

Healthy Start replaced the Welfare Food scheme, which targeted the same households, but provided one token each week that could be exchanged for seven pints of milk. Healthy Start replaced the Welfare Food scheme, which was introduced in the 1940s. This operated similarly to Healthy Start, targeting low-income families, but vouchers could only be used to purchase milk. The Healthy Start scheme therefore explicitly intended to promote healthy diets by increasing fruit and vegetable consumption, following recommendations made by the UK Committee on Medical Aspects of Food and Nutrition Policy (Department of Health, 2002) and the WHO, 1990, WHO, 2003.^6^ Table 1 compares the key features of the scheme. Further information is given in Appendix A, where we also discuss how we deal with the previous scheme in our analysis. The focus of our analysis is spending on fruit and vegetables. Supported by the data, we assume that milk is an essential good for families with young children and is consumed in relatively fixed quantities, depending on family size. Furthermore, we consider the policy to be neutral with respect to the purchase of formula milk, since the Healthy Start scheme provides additional vouchers to households with a child aged less than one to compensate them for the loss of tokens for formula milk. The qualitative predictions of the effect of the Healthy Start Vouchers are therefore assumed to be unaffected. In Appendix A, we show using a difference-in-difference approach that spending on milk did not change differentially for eligible versus ineligible households, after compared to before the reform, and our results are robust to including milk spending with spending on fruit and vegetables. However, the *net* value of Healthy Start Vouchers is less than a situation with no pre-existing scheme. In other words, when we estimate the marginal propensity to consume out of one £ of vouchers, we will underestimate the true response. We attempt to deal with this in Section 5.5, where we estimate the ‘true’ value of the vouchers. One other important aspect of the change from the Welfare Food scheme to the Healthy Start scheme is that households with children aged three or over became ineligible. In principle, this could suggest a (difference in) regression discontinuity design to identification, but we lack sufficient observations to identify the effects of the reform precisely using this approach and instead adopt a difference-in-differences approach, which we describe in more detail in Section 4.

**Table 1:** Comparison of the Welfare Food scheme and the Healthy Start scheme.

	Welfare Food scheme	Healthy Start scheme
Families on benefits receive:	One voucher per family with children aged ≤4	One voucher per pregnant woman, one voucher per child aged ≤3 (two vouchers per infant aged 0–1)
The value per voucher:	Approximately £2.80 a	£2.80 from 27 November 2006
		£3.00 from 6 April 2008
Vouchers can be spent on:	7 pints of cows’ milk (or 900 g of formula for infants aged 0–1)	Milk, plain fresh fruit and vegetables

*Notes*: Both schemes apply to households who receive Income Support, Income-based Jobseeker’s Allowance, or Child Tax Credit with an income below a certain year-specific threshold (£13,230 in 2003/04, £13,480 in 2004/05, £13,910 in 2005/06, £14,155 in 2006/07, £14,495 in 2007/08, £15,575 in 2008/09).

a The value of a voucher during the Welfare Food scheme depends on the price of milk, as each voucher was exchangeable for 7 pints of cow’s milk. In 2006, the price of a pint of cow’s milk was approximately 40p, so 7 pints is equal to approximately £2.80.

### 2.2 Effects on spending and nutrients

The standard economic incentive effects of targeted benefits such as the Healthy Start scheme predict that they have the greatest positive effect on spending on the targeted good for “distorted households”, i.e. those who would spend less than the value of the vouchers on the targeted goods if they were given an equivalent-value cash benefit (Southworth, 1945). For “infra-marginal households”, who would spend at least the value of the voucher on the targeted good if they were given cash, the standard economic incentive effect of the vouchers is the same as cash benefits. Infra-marginal households can use the voucher to cover existing spending and then re-allocate their (non-voucher) expenditure among other goods (see Appendix A for further discussion and diagrammatic illustration).

The insights from this standard model frame our main empirical investigation. First, it is an empirical question whether targeted benefits have a positive effect on spending on the targeted good (fruit and vegetables). If most households are infra-marginal, the vouchers may lead to increased spending on other items, even unhealthy food. We therefore look at whether the introduction of the vouchers increased spending on fresh fruit and vegetables and whether any increase in spending was greater than for an equivalent value cash benefit.

Second, we take account of possible indirect effects. In the context of a policy that is aimed at improving the health and diets of low-income households with young children (Lucas et al., 2013), it is important to take a broader perspective. As discussed in Cunha (2014), households that increase spending on (fresh) fruit and vegetables may decrease spending on close substitutes, including other healthy alternatives such as frozen fruit and vegetables or pulses. To assess the overall effect of the program, it is not enough just to show that spending on fresh fruit and vegetables increases, but it is also important to look at evidence on the outcomes that policy-makers are trying to change. We do not observe health outcomes for households, but we can measure the nutrient content of foods brought into the house. We do this in Section 4.2, where we look at the effect of the program on purchases of nutrients defined over the entire range of foods in households’ shopping baskets. Our analysis focuses on nutrients known to be beneficial for child development; we also look at nutrients where current levels of consumption are well above recommended levels, such as sugars and saturated fats.

## 3 Data

We use data on all grocery purchases brought into the home made by a rolling panel of households in the UK over the period December 2004–November 2008, a period that runs two years prior to the introduction of the scheme (December 2004–November 2006) and two years after the scheme was introduced (December 2006–November 2008). The full data for this period contains 6235 households and 49,207 household-month observations.^7^ Column 1 of Table 2 presents some descriptive statistics of this full sample, with panel A showing descriptive statistics for the period prior to the introduction of the scheme, and Panel B for the period after the scheme was introduced. We can see that households spent an average of £19.26 per month on fruit and vegetables prior to the introduction of the scheme, which increased to £20.77 afterwards. Only 3% of households purchase at least five portions of fruit and vegetables per person per day prior to the introduction of the scheme, which reduced to 2% for the post-scheme period.

**Table 2:** Means and standard deviations of the Kantar data, by eligibility.

	(1)		(2)		(3)		(4)	
	Full sample		Estimation sample		Eligible		Ineligible	
	Mean	SD	Mean	SD	Mean	SD	Mean	SD
**Pre**								
Total spending (£): fruit & vegetables	19.26	(13.0)	16.49	(11.8)	17.62	(11.6)	14.82	(11.8)
Total quantity (kg): fruit & vegetables	17.86	(11.0)	16.37	(10.4)	17.39	(10.0)	14.87	(10.8)
Prop. purchase ≥5 portions pppd	0.03	(0.2)	0.02	(0.2)	0.02	(0.1)	0.03	(0.2)
Total spending: all foods	178.6	(72.1)	176.8	(68.1)	181.6	(67.2)	169.7	(68.9)
Number of vouchers eligible for	N/A		N/A		N/A		N/A	
Value of vouchers eligible for	N/A		N/A		N/A		N/A	
Household size	3.69	(0.9)	3.67	(1.0)	3.84	(0.9)	3.40	(1.0)
≥3 months pregnant	0.05	(0.2)	0.06	(0.2)	0.09	(0.3)	–	–
No. of 0 year olds	0.17	(0.3)	0.14	(0.3)	0.24	(0.4)	–	–
No. of 1–3 year olds	0.52	(0.6)	0.53	(0.6)	0.88	(0.5)	–	–
No. of 4 year olds	0.18	(0.4)	0.20	(0.4)	0.14	(0.3)	0.31	(0.4)
No. of 5–18 year olds	0.92	(0.9)	1.08	(1.0)	0.84	(0.9)	1.44	(0.9)
No. of adults	1.97	(0.4)	1.84	(0.6)	1.90	(0.5)	1.75	(0.6)
No. of households	4038		296		197		144	
No. of household-month observations	21081		2593		1541		1052	

**Post**								
Total spending (£): fruit & vegetables	20.77	(13.8)	18.43	(12.1)	19.83	(12.0)	17.47	(12.1)
Total quantity (kg): fruit & vegetables	17.03	(01.4)	16.80	(10.0)	18.19	(10.1)	15.85	(9.8)
Prop. purchase ≥5 portions pppd	0.02	(0.1)	0.01	(0.1)	0.01	(0.1)	0.02	(0.1)
Total spending: all foods	202.2	(78.7)	195.6	(73.6)	199.0	(69.9)	193.3	(76.0)
Number of vouchers eligible for	0.83	(0.9)	0.55	(0.8)	1.35	(0.6)	0.00	(0.00)
Value of vouchers eligible for	10.17	(11.4)	6.72	(9.5)	16.5	(7.8)	0.00	(0.00)
Household size	3.77	(0.9)	3.73	(1.0)	4.09	(0.8)	3.44	(1.0)
≥3 months pregnant	0.04	(0.2)	0.04	(0.2)	0.09	(0.3)	–	–
No. of 0 year olds	0.09	(0.3)	0.09	(0.3)	0.19	(0.4)	–	–
No. of 1–3 year olds	0.47	(0.6)	0.42	(0.6)	0.93	(0.5)	–	–
No. of 4 year olds	0.18	(0.4)	0.19	(0.4)	0.12	(0.3)	0.25	(0.4)
No. of 5–18 year olds	1.13	(1.0)	1.35	(1.0)	1.07	(1.0)	1.58	(0.9)
No. of adults	1.99	(0.4)	1.86	(0.6)	2.00	(0.5)	1.75	(0.6)
No. of households	4850		296		161		192	
No. of household-month observations	28126		2383		969		1414	

*Note*: The full sample includes all household-months in the Kantar data, excluding those with periods of non-recording longer than seven days, outliers in spending and quantity purchased, and households in the South West. The estimation sample are those observed both before and after the introduction of the scheme, predicted to be on benefits (i.e. both the head and spouse working 8 h or less, or unemployed), and have at least one child aged 8 or less or are pregnant at some point during the period December 2004–November 2008. Eligible households are those ≥3 months pregnant, or with a child aged 0–3; Ineligible households are those with children aged 4–8, or not yet pregnant. The number of eligible and ineligible households exceeds the total number of households, as eligibility is time-varying, so households can be eligible in one month and ineligible in another. A portion of fruit and vegetables is defined as 80 g.

The data are collected by the market research firm Kantar as part of their Worldpanel; they are similar in nature to the Nielsen Homescan data that are commonly used to study US consumer purchases. Purchases are recorded at the individual transaction level using a handheld scanner in the home. The advantages of these data are that they are longitudinal, households typically remain in the sample for several years, and they provide very detailed data on the specific food products that households purchase, along with detailed demographic information. Standard consumer surveys, such as the Expenditure and Food Survey in the UK or the Consumer Expenditure Survey in the US, are cross-sectional and do not record information at such a disaggregate level. In addition, the UK Expenditure and Food Survey does not record purchases that are made with Healthy Start Vouchers and Welfare Food Tokens, whereas the Kantar data does.^8^ The Kantar data include rich demographic information, including the month of birth of all household members. This allows us to identify which households are eligible for vouchers based on the exact age and presence of children. The detailed product information allows us to precisely identify which products can be purchased with the vouchers, and the transaction level data allows us to accurately identify the timing of purchases.

In the UK approximately 28% of all fruit and vegetable purchases consist of loose produce, the other 72% is sold pre-packaged. One complicating factor is that around 20% of household-month observations are for households that are only asked to scan packaged items.^9^ We deal with this issue in two ways. First, all our analyses include household fixed effects, exploiting within-household changes in fruit and vegetables spending. As the requirement to scan loose items does not vary within a household, any differences in levels of spending are captured in the fixed effects.^10^ Second, to ensure that this is not affecting our results, we exclude the 20% of observations that do not record loose fruit and vegetables in the robustness checks; our results are robust to these concerns.

There are important advantages of using these data, and they increasingly being used in social science research (see, for example, Aguiar and Hurst (2007) and Dubois et al. (2014), but as with all data, there are potential limitations. There are issues that are common with all survey data, such as the fact that it is difficult to attract some demographics groups, in particular single young males. An additional concern with longitudinal data is that participants might suffer from fatigue bias, and that over time their reporting might become less accurate. This is an issue that Kantar themselves are concerned with, and they take considerable effort to monitor participants and remove them from the panel if them if they believe that this is a problem.

Leicester and Oldfield (2009) and Griffith and O’Connell (2009) provide detailed studies of the quality of the Kantar data, and compare it to other data sources (a related analysis is carried out for the US data by Einav et al. (2010). In summary, they show that, if you condition on a household regularly reporting expenditure on a range of grocery products, as we do, then the Kantar data follow the patterns and trends seen in other data sources.

Leicester and Oldfield (2009) carry out detailed analysis of the extent of attrition, fatigue bias and reporting error in these data. They compare the Kantar data to the Living Cost and Food Survey, which is the other standard data source on food purchases in the UK. They conclude that attrition rates and fatigue in recording is generally quite low. Griffith and O’Connell (2009) provide a detailed description of the nutritional information; they show that the product level information provides significantly more information than the more aggregated product level nutritional information available in the Living Cost and Food Survey or in the National Diet and Nutrition Survey (the main UK data source recording nutritional intake data), and that the Kantar data avoids the well known problem of underreporting in intake surveys (see, for example, Briefel et al., 1997 and Rennie et al., 2007).

One specific limitation of the data for our purposes is that we do not directly observe whether the household receives means-tested benefits (including the Vouchers), nor do we observe complete information on household income. We exploit the fact that the receipt of benefits in the UK is a function of the number of hours worked: benefits are only available to individuals who work less than 16 h a week with a partner who is working less than 24 h a week. The employment status of the head of household as well as the spouse is well recorded in the Kantar data in the following categories, which we observe each year: not working, unemployed, in education, working less than 8 h a week, working between 8 and 29 h, and working 30 or more hours a week. These bands unfortunately do not allow us to identify individuals who work less than 16 or less than 24 h a week. To maximise the likelihood that the households in our sample receive benefits, we therefore define households that are likely to be on benefits as those where both the head and spouse work less than 8 h a week, or are unemployed. Our empirical analysis focuses on this set of households only.

To assess how well our simple rule does in predicting which households are on benefits, we look at data from the Expenditure and Food Survey (EFS), which contains both hours worked and actual benefit receipt. We do a very good job in correctly assigning households that are in receipt of benefits. Among the households that we predict to be on benefits, 91.7% actually did receive benefits. Hence, we are likely to have very few false positives; Table B1 in Appendix B provides further details. However, some households on benefits are not captured by our definition (i.e. we have a higher rate of false negatives). Using only hours worked, we identify 68.3% of all households who are actually on benefits. There are some selection effects among the households that we do capture: compared to the (representative) EFS sample that receive benefits, they are more likely to have a head who is not in work and not married. In addition, spending on milk, fruit and vegetables amongst the households that we do identify as being on benefits is lower than amongst those households that are on benefits but which we do not capture. Hence, our analyses are based on households that are more likely to be unemployed and unmarried, and the results may be less generalisable to the full population of benefit recipients.

We observe hours of work each year, and hence, a household’s benefit status may change over time. This is particularly relevant for households with young children, who may be more likely to change employment status or hours worked within a relatively short time span. As changes in benefit status might affect household shopping behaviour, for example, due to differences in the availability of time, in our main analysis we focus on households that are *always* on benefits. We examine the sensitivity of our approach in the robustness analysis to including households that come on and off benefits, and find that our results are robust.

In a second robustness check we take an approach similar to Arellano and Meghir (1992); we use a wider set of characteristics that are available in both the Kantar and in the EFS to predict the probability of being on benefits. We use this probability in two ways. First, to define a discrete group of households that are on benefits, and second, to use the probability of being on benefits as a weight in an analysis that includes all households, similar to a propensity score.

Our sample includes 296 households (4976 household-months) in the Kantar panel that are observed both before and after the introduction of the scheme, predicted to be on benefits (i.e. both the head and spouse working 8 h or less, or unemployed), and have at least one child aged 8 or less or are pregnant at some point during the period December 2004–November 2008.^11^ This is after dropping a small number of outliers, defined as observations in the top percentile of the expenditure and quantity distribution, and those that have periods of non-recording longer than seven days. Eligible households are defined as those with children aged 0–3, or where the woman is at least three months pregnant. Households that work similar hours but with children aged 4–8, or in the period just before the woman is pregnant, act as the control group of ineligible households. Based on the age and presence of children, 50% of the household-month observations are eligible for Healthy Start Vouchers. The majority of these (68%) are eligible for one voucher; 25% are eligible for two, and 7% three or more.

We exploit the panel nature of our data and use information on households’ spending on fruit, vegetables and milk prior to the introduction of Healthy Start scheme to identify which households are likely to be distorted and infra-marginal. We consider only households who are observed at least four months prior to the reform and identify distorted households as those who spent less than the value of the vouchers on milk, fruit and vegetables per 0–8 year old child at any time prior to the introduction of the scheme, while infra-marginal households are those who never spent less than that amount on milk, fruit and vegetables per child. Characteristics for the two groups of households are summarized in Appendix C, Table C1. Based on household average spending prior to the introduction of the scheme, 62% of households are likely to be distorted, and 38% are likely to be infra-marginal. Based on standard economic theory, the vouchers are likely to increase spending on fruit and vegetables among distorted household by more than a cash alternative. However, while monthly average spending on fruit and vegetables is lower among distorted consumers, there is only a small difference between their level of spending and the level of the vouchers, indicating that many distorted households only need to increase their spending by a small amount to reach the ‘kink’ (the value of the voucher).

Table 2 columns (2), (3), and (4), present the means and standard deviations of a set of characteristics for households in our sample, which are all estimated to be on benefits. In other words, this only includes the 296 households (or 4976 household-months) observed both before and after the introduction of the scheme, predicted to be on benefits, and who have at least one child aged 8 or less or are pregnant at some point during the period December 2004 to November 2008. Column 2 presents the statistics for this estimation sample; column 3 for eligible households, and column 4 for ineligible households. Panel A refers to the pre-scheme period, whilst Panel B shows the descriptives for the post-scheme period.

We start with monthly spending on different foods, including fruit and vegetables. There are small differences between eligible (with young children) and ineligible households (with older children); the group of eligibles tend to spend more on fruit and vegetables, as well as on all foods together, both before and after the introduction of the scheme. Looking at household size, we see that they are also slightly larger than ineligible households. In our analysis, we control for household fixed effects, and therefore only identify the effects of the scheme from changes *within* households. We also account for time-varying household characteristics, including the number of adults and children in the household, and a second order polynomial in the age (in months) of the youngest and oldest child in the household.

Eligibility is defined purely in terms of exogenous characteristics of the households, namely the age of children. However, there may be some concern that the eligibility threshold is associated with discrete changes in behaviour, for example, because children start school or nursery. The benefit of our difference-in-differences strategy is that we control for any such effects so long as they are common before and after the introduction of the scheme. Our identifying assumption is that, absent the reform, spending among eligible households would have evolved in the same way as spending among the ineligible households. In Appendix C, we test the robustness of the common trends assumption by means of a placebo test, specifying a ‘placebo reform’ as one introduced in November 2005 (one year prior to the true start of the scheme) and restrict the data to December 2004 to November 2006. We find no effect on fruit and vegetables spending or quantity out of placebo vouchers, suggesting the common trend assumption holds (Table C2, columns (1) and (2)).

## 4 Empirical strategy and main results

### 4.1 Effect on spending on fruit and vegetables

We start with a binary “treatment effect” specification that tests whether the reform led to a significant increase in spending on fruit and vegetables:

(1) $$FV_{ht}=\beta_{0}+\beta_{1}Post_{t}+\left(\beta_{2}+\beta_{3}Post_{t}\right)E_{ht}+\delta X_{\mathrm{ht}}+\phi_{h}+\tau_{t}+e_{ht}$$

where *FV*_*ht*_ is expenditure on fruit and vegetables (in £) for household *h* in month *t*. *Post*_*t*_ is a binary indicator for the months after November 2006, when the scheme was introduced. *E*_*ht*_ is a binary indicator for whether the household is eligible for Healthy Start Vouchers, defined before and after the reform and based on the presence and age of children in the household. Interacting *Post*_*t*_ and *E*_*ht*_ captures the overall effect of the reform. The vector **X**_*ht*_ includes other time-varying household-level covariates, including a full set of fixed effects for the number of children and number of adults in the family to control for varying food needs across households (Currie, 2003). We also control flexibly for the age of the youngest and the oldest child (in months). Household fixed effects *ϕ*_*h*_ control for time invariant differences in preferences across households, and year and month effects, *τ*_*t*_, pick up common annual and seasonal fluctuations in spending. *e*_*ht*_ is an idiosyncratic error, clustered by household.

Following Moffitt (1989) and Hoynes and Schanzenbach (2009), we also test for equality of responses to the vouchers and an increase in the grocery budget using the following specification:

(2) $$FV_{ht}=\beta_{0}+\beta_{1}Post_{t}+\left(\beta_{2}+\beta_{3}Post_{t}\right)Value_{ht}+\theta Y_{ht}^{}+\delta X_{ht}+\phi_{h}+\tau_{t}+e_{ht}$$

where *Value*_*ht*_ denotes the value of the Healthy Start Vouchers (in £) for which the household is eligible (based on the age and number of children and defined before and after the reform) and *Y*_*ht*_ is the value of total household grocery expenditure (in £). Total grocery spending is spending on food and other fast moving consumer goods such as toiletries and household goods.^12^ The marginal propensity to consume (MPC) fruit and vegetables out of grocery spending is captured by *θ*. Since our measure of total grocery spending includes the value of the vouchers, the parameter *β*_3_ captures the difference in the MPC out of vouchers compared to grocery spending, while the overall MPC out of vouchers is equal to *θ* + *β*_3_.

Results are presented in Table 3. Column (1) shows that the reform led to a sizeable increase in spending on fruit and vegetables among eligible households compared to the ineligible control group. The vouchers led to an estimated increase in spending of £2.43 a month, an increase of around 15 per cent compared to mean, pre-reform spending of £16.49.

**Table 3:** The effect of Healthy Start Vouchers on expenditures and quantity purchased.

Dependent variable:	(1)	(2)	(3)	(4)
	F&V expenditures (in £)		F&V quantity (in kg)	
Treatment effect (binary), β_3_ in Eq. (1)	2.425***		1.789***	
	(0.643)		(0.647)	
Treatment effect (per £), β_3_ in Eq. (2)		0.082***		0.080***
		(0.029)		(0.026)
Household is eligible, β_2_ in Eq. (1)	−2.659***		−1.762**	
	(0.757)		(0.698)	
Post reform, β_1_	−0.671	−0.581	−0.460	−0.631
	(0.820)	(0.731)	(0.898)	(0.794)
Value of voucher (in £), β_2_ in Eq. (2)		−0.101***		−0.067**
		(0.034)		(0.031)
Total grocery spending (in £), θ in Eq. (2)		0.062***		0.052***
		(0.003)		(0.003)

MPC out of vouchers		0.144***		0.132***
		(0.285)		(0.026)

Mean F&V spending pre-scheme	16.49	16.49	16.37	16.37
Sample mean of value of vouchers	8.53	8.53	8.53	8.53
Number of households	296	296	296	296
Number of household-months	4976	4976	4976	4976

*Notes*: Column (1) and (3) show esimates of the coefficients from Eq. (1), columns (2) and (4) show estimates of the coefficients from Eq. (2). The data cover the period between December 2004–November 2008. All columns include household, month and year fixed effects, age and age squared of youngest and oldest child (in months), dummies for whether household includes: 2 adults, 3+ adults, 1 child, 2 children, 3 children, 4+ children, and a dummy indicating whether the household did not buy any fruit and vegetables that month. Eligible households are those with a child aged 0–3 or where the woman is ≥3 months pregnant. The post-reform period refers to December 2006 onwards. MPC stands for marginal propensity to consume. F&V stands for fruit and vegetables. Robust standard errors in parentheses, clustered by household. * p < 0.10, ** p < 0.05, *** p < 0.01.

Column (2) presents our estimates of the marginal propensity to consume fruit and vegetables out of both grocery spending and Healthy Start Vouchers. The results show that the vouchers have a stronger effect on fruit and vegetables spending than would an equivalent value cash benefit (i.e. we can reject that *β*_3_ = 0). The estimated MPC out of vouchers is 0.144 (±0.285); in other words, each additional £1 of Healthy Start vouchers caused households to increase their spending on fresh fruit and vegetables by 14 pence.

In column (3) we report results with the *quantity* of fruit and vegetables purchased as the outcome variable. Given the policy objective to improve diets, it is important to show a quantity response because households could have increased expenditure, for example, through shopping around less and buying more expensive fruit and vegetables, rather than increasing the quantity. Our results indicate an increase of 1.79 kg per month following the introduction of the reform, confirming that the introduction of the scheme was associated with an increase in the amount of fruit and vegetables brought into the home of eligible households.

### 4.2 Effect on nutrient composition of households' shopping baskets

As discussed, the Healthy Start scheme may have caused households to reduce spending on healthy substitutes and also to increase spending on other (unhealthy) food items, potentially offsetting any positive effect on spending on fruit and vegetables. To assess the overall nutritional impact of the programme, we study its effect on the nutrient content of households’ total shopping basket, focusing on a number of key nutrients.

We look at a set of nutrients known to be important for child development, including fibre, beta-carotene (vitamin A), Vitamins C, D and E, potassium, iron and zinc (British Nutrition Foundation, 2016a, British Nutrition Foundation, 2016b; WHO, 2011, WHO, 2012), a set of nutrients that are generally considered to be less healthy, and of which many households consume more than recommended (saturated fats and added sugars), and also carbohydrates, protein and total calories. We use information collected and published by the Department for Environment and Rural Affairs (DEFRA) for use with the EFS data on the nutritional composition of 249 food groups, and match this to products in the Kantar data.^13^ Evaluating the impact of the policy on total nutrients purchased provides a useful way to aggregate spending into meaningful categories that are directly relevant to the intended impact of the policy, which was to improve the overall nutritional quality of households’ shopping baskets.

Table 4 shows these results. The 20 columns show the estimated coefficients from regressions of the forms of Eq. (1). We see that the amount of fibre, beta-carotene (vitamin A), carbohydrates and iron increase. These is weak evidence (at the 10% significance level) that calories, potassium, and zinc also increase. There is no evidence that levels of other nutrients change, and most notably there is no evidence that any of the less healthy nutrients (non-milk extrinsic (i.e. added) sugars, saturated fats) increase. These results suggest that the overall effects of the Health Start scheme were to improve the nutritional *quality* of eligible households' shopping baskets both in terms of the quantity of fruit and vegetables and in terms of the overall nutrient composition. However, there is also evidence that it might have been associated with an increase in total calories.

**Table 4:** The effect of Healthy Start Vouchers on total nutrient purchases.

	(1)	(2)	(3)	(4)	(5)	(6)	(7)	(8)	(9)	(10)
Dependent variable:	Fibre (Southgate)	Fibre (Englyst)	Beta-carotene	Vitamin C	Vitamin D	Vitamin E	Kcal	Carbo-hydrates	Total sugars	Non-milk extrinsic sugars
*Unit of measurement*	*g*	*g*	*μg*	*mg*	*μg*	*mg*	*kcal*	*g*	*g*	*g*
Treatment effect	82.4^***^	63.9^***^	8390.8^**^	110.6	6.1	34.8	5460.3^*^	796.7^**^	376.2	243.5
	(30)	(22)	(3239)	(199)	(6)	(22)	(2994)	(395)	(232)	(187)
Household is eligible	−33.2	−28.3	−4486.3	−195.6	5.1	9.9	493.8	−209.0	−146.7	−86.7
	(29)	(21)	(3193)	(204)	(6)	(20)	(2597)	(351)	(209)	(167)
Mean pre-scheme										
nutrient purchase	1311	974	107910	5277	198	788	137077	17779	9245	6476
% Change	6.3	6.6	7.8	2.1	3.1	4.4	4.0	4.5	4.1	3.8

	(11)	(12)	(13)	(14)	(15)	(16)	(17)	(18)	(19)	(20)
Dependent variable:	Fats	Saturated fats	Mono-unsaturated fats	Poly-unsaturated fats	Sodium	Protein	Potassium	Iron	Zinc	Calcium
*Unit of measurement*	*g*	*g*	*g*	*g*	*mg*	*g*	*mg*	*mg*	*mg*	*mg*
Treatment effect	206.7	67.9	79.8	43.0	6.0	159.9	8.5^*^	35.8^**^	21.6^*^	1389.4
	(138)	(47)	(55)	(32)	(5)	(108)	(5)	(17)	(13)	(1435)
Household is eligible	102.9	28.5	40.8	29.8	0.5	13.4	−4.9	−7.1	−0.5	677.5
	(121)	(43)	(48)	(29)	(5)	(97)	(4)	(15)	(11)	(1354)
Mean pre-scheme										
nutrient purchase	5524	2049	2090	1024	212	4640	214	755	556	60264
% Change	3.7	3.3	3.8	4.2	2.8	3.4	4.0	4.7	3.9	2.3

No. of households	296	296	296	296	296	296	296	296	296	296
No. of household-months	4976	4976	4976	4976	4976	4976	4976	4976	4976	4976

*Notes*: Observation period runs from December 2004 − November 2008. All columns include household, month and year fixed effects, age and age squared of youngest and oldest child (in months), dummies for whether household includes: 2 adults, 3+ adults, 1 child, 2 children, 3 children, 4+ children, and a dummy indicating whether the household did not buy any fruit and vegetables that month. Eligible households are those with a child aged 0–3 or where the woman is ≥3 months pregnant. The post-reform period refers to December 2006 onwards. Robust standard errors in parentheses, clustered by household. * p < 0.10, ** p < 0.05, *** p < 0.01.

We investigate whether the Healthy Start scheme also affected the proportion of households that meet their Reference Intake for a set of nutrients that are considered important for child development, including protein, vitamin C, potassium, iron, zinc, and calcium. Reference Intakes (previously known as Guideline Daily Amounts, or GDAs) give an indication of how much of each nutrient the average person needs; they vary by age and gender (see Department for Environment, Food and Rural Affairs, 2015). We calculate an index of whether a household is meeting this Reference Intake level on average over a month by summing the individual household member’s Reference Intake for a month. We create an indicator that equals 1 when the household’s purchases of that nutrient equals or exceeds the household’s monthly Reference Intake, and 0 if it is below the Reference Intake. Note that this indicator is based on household *purchases*, not *consumption*. We use this as the dependent variable in regressions of the forms of Eq. (1).

Table 5 shows a positive treatment effect for iron and potassium that is significantly different from zero at the 10% level. The probability that households exceed their Reference Intake for iron and potassium increases by 4.5 and 5.2 percentage points respectively. Prior to the introduction of the Scheme 10.2% and 23.1% of households exceeding their Reference Intakes for iron and potassium so this represents a sizeable increase. These results suggest that the Health Start scheme improved the dietary quality of eligible households' shopping baskets, bringing them closer to the recommended Reference Intakes.

**Table 5:** The effect of Healthy Start Vouchers on the proportion of households exceeding their monthly Reference Intakes.

	(1)	(2)	(3)	(4)	(5)	(6)
	Protein (g)	Iron (mg)	Zinc (mg)	Potassium (g)	Vitamin C (μg)	Calcium (mg)
Treatment effect	0.027	0.045^*^	0.020	0.052^*^	−0.003	0.051
	(0.04)	(0.02)	(0.02)	(0.03)	(0.03)	(0.03)
Eligible	−0.006	−0.052^**^	0.046^*^	−0.029	−0.020	0.005
	(0.03)	(0.02)	(0.02)	(0.03)	(0.03)	(0.03)
Monthly average of nutrient purchased	4708	769	564	216	5292	60853
Reference Intake (RI)	4098	1137	817	283	3961	70964
Mean pre-scheme proportion exceeding						
their monthly RI	0.660	0.102	0.131	0.231	0.644	0.358
% Change	5.1	44.1	15.3	22.5	−0.5	14.2
Number of households	296	296	296	296	296	296
Number of household-months	4976	4976	4976	4976	4976	4976

*Notes*: ‘Monthly average of nutrient purchased’ denotes the average monthly amount of each nutrient purchased by households; ‘Reference Intake (RI)’ indicates the mean monthly RI, i.e. what households ‘should’ have purchased based on the number, age and gender of household members. The observation period runs from December 2004–November 2008. All columns include household, month and year fixed effects, age and age squared of youngest and oldest child (in months), dummies for whether household includes: 2 adults, 3+ adults, 1 child, 2 children, 3 children, 4+ children, and a dummy indicating whether the household did not buy any fruit and vegetables that month. Eligible households are those with a child aged 0–3 or where the woman is ≥3 months pregnant. The post-reform period refers to December 2006 onwards. Robust standard errors in parentheses, clustered by household. * p < 0.10, ** p < 0.05, *** p < 0.01.

## 5 Further analysis

In this section, we conduct some additional analyses. First, we look at treatment effect heterogeneity across distorted and infra-marginal households. Second, we explore potential spillover effects on other purchases. Third, we show that our main analysis is robust to alternative specifications, including functional form, the set of foods considered, the sample of households used, the definition of benefit receipt.

### 5.1 Treatment effect heterogeneity

We extend the analysis of the effects of the scheme to consider separately responses among distorted and infra-marginal consumers. Our aim is to shed light on the mechanisms through which the scheme worked to increase spending on fruit and vegetables. “Labelling effects” in relation to benefits (the notion that the labels attached to cash benefits can affect the way they are spent) and the role of the health professionals, are potential mechanisms through which Healthy Start vouchers may have affected spending. An empirical test for the presence of these behavioural effects, as opposed to standard economic incentives, is whether the effect of the vouchers is greater than that of equivalent cash for both distorted consumers and infra-marginal consumers (consistent with behavioural effects) or whether the effect of the vouchers is greater than that of equivalent cash just for distorted consumers (consistent with standard economic incentives).^14^

We re-run our binary treatment effect specification allowing the effect of the reform to vary by whether households are distorted (D) or infra-marginal (IM). The definition of the two groups is discussed above and is based on pre-reform levels of spending.

(3) $$FV_{ht}=\beta_{0}+\beta_{1}Post_{t}+\left(\beta_{2}+\beta_{3}Post_{t}\right)E_{ht}^{D}+\left(\beta_{4}+\beta_{5}Post_{t}\right)E_{ht}^{IM}+\delta X_{ht}+\phi_{h}+\tau_{t}+e_{ht}$$

The results, reported in Column (1) of Table C3, Appendix C, show that there was a significant increase in spending on fruit and vegetables among distorted consumers (equal to £2.83 per month) and no change among infra-marginal consumers. We find a similar pattern for quantities purchased.

We also test for the equality of responses to vouchers and an increase in the household’s grocery budget separately among the two groups using the following specification:

(4) $$FV_{ht}=\beta_{0}+\beta_{1}Post_{t}+\left(\beta_{2}+\beta_{3}Post_{t}\right)E_{ht}^{D}Value_{ht}+\left(\beta_{4}+\beta_{5}Post_{t}\right)E_{ht}^{IM}Value_{ht}+\theta^{D}Y_{ht}^{D}+\theta^{IM}Y_{ht}^{IM}+\delta X_{ht}+\phi_{h}+\tau_{t}+e_{ht}$$

Using this specification, we can directly test the predictions from the standard model that the MPC out of the vouchers will be greater than the MPC out of grocery spending for distorted consumers (i.e. *β*_3_ > 0) while the MPC out of the vouchers will be the same as the MPC out of grocery spending for infra-marginal consumers (i.e. *β*_5_ = 0). We allow the effect of grocery spending (*Y*_*ht*_) to vary across distorted and infra-marginal consumers.

The results are reported in column 2 of Table C3, Appendix C. They are robust to using the quantity of fruit and vegetables purchased as the dependent variable (shown in column (4)). We find that the vouchers have a significantly greater effect than grocery spending among distorted consumers, i.e. we can reject that β_3_ = 0. However, we cannot reject that the vouchers are equivalent to cash benefits for infra-marginal consumers, i.e. we cannot reject that β_5_ = 0. These effects are in line with the predictions from standard economic theory and do not indicate a role for labelling or other behavioural effects.

### 5.2 Spillover effects

We explore whether there were any spillover effects, including on unhealthy foods (e.g. prepared foods, alcohol), as well as close substitutes to fresh fruit and vegetables (e.g. fruit juices, frozen fruit and vegetables).

First, we analyse the effects of the scheme on similar foods that were not allowed to be purchased with the vouchers. Columns (1) of Table C4 presents the effects on spending on fruit juice, where we see a weak *negative* response for both outcomes. Column (2) shows the effect on frozen fruit and vegetables, which was also not allowed to be purchased with the vouchers, where there is no significant response.

Columns (3) to (14) show the effects of the scheme on other food groups, including prepared sweet foods (e.g. biscuits, cakes), prepared savoury foods (e.g. snacks, popcorn), crisps, non-diet and diet drinks, grains, dairy, cheese, red meats, poultry/fish, alcohol and non-foods. We find no significant effects on any of these groups, which also holds when we estimate a binary treatment effect (as in Eq. (1)). This suggests that households did not reallocate their spending to other (unhealthy or healthy) food categories, and that the increase in spending was limited to items of spending that the vouchers could be spent on.

### 5.3 Functional form and endogeneity of total grocery expenditure

We test the robustness of our results to potential functional form concerns.

One possible concern is whether our results are sensitive to whether expenditure and quantity are measured in levels of logs. In Table C5 in Appendix C we replicate the main results shown in Table 3 using logs instead of levels. Using logs in the specifications that distinguish between distorted and infra-marginal households also give similar results (available upon request), indicating that the main predictions from theory hold across all specifications. Below, we also show that the results hold when we specify fruit and vegetable spending as a share of total spending (Column (4) of Table C6, Appendix C).

Another possible concern about functional form is that estimation of the MPC out of grocery spending depends on how the latter is included in the regression. Our main specification controls linearly for total spending on foods and fast moving consumer goods. If instead we also include a quadratic term in total grocery expenditure this is statistically significant at the 1% level, but has little impact on our conclusions.

We might also be concerned that grocery spending is endogenous; times of high overall expenditures might be correlated with times of low or high fruit and vegetable expenditures. To investigate whether this is a concern, we re-estimate the model using total *food* spending (excluding expenditure on toiletries, cleaning products and other household items); this is valid under the assumption that food spending is separable from expenditure on these other items. We then instrument food spending using expenditure on these other items. Although this increases our estimate of the MPC out of grocery spending, it has little impact on our conclusions. These results are robust across the different specifications (available from the authors upon request).

### 5.4 Loose fruit and vegetables

As discussed in Section 3, approximately 20% of our sample do not scan items without a barcode, such as loose fruit and vegetables, or meat and fish purchased over the counter. We include household fixed effects in all regressions, so we do not expect this to have an impact on our results. However, to check this we re-run our analyses using the sample of households that do scan loose items. This is presented in column (2) of Table C6, showing very similar estimates to those obtained in the full sample.

### 5.5 Estimating the ‘true’ value of the vouchers

Due to the pre-existing Welfare Food scheme that provided households with milk tokens, the net change in value from the introduction of the Healthy Start vouchers is less than a situation without the pre-existing scheme. This implies that our estimates of the MPC out of vouchers are likely to *underestimate* the true response. We argue that households consume milk in relatively fixed quantities, depending on household size. Assuming that milk is separable from other food spending, conditional on household size, we can approximate the value of the voucher for fruit and vegetables *net* of what households spend on milk, and more precisely estimating the marginal propensity to consume fruit and vegetables out of vouchers. We do this by subtracting households’ milk spending from the total value of the vouchers they are eligible for, and comparing the MPC out of vouchers to that out of grocery expenditure using this new definition.

Column (3) in Table C6 presents the results, showing larger estimates than for our main estimates: households additionally increase their fruit and vegetable expenditures by £0.09 per £ of vouchers (compared to a £ increase in the grocery budget).

### 5.6 Benefit recipients

We next explore the robustness of our analysis to different ways of defining benefit receipt. Our main specification uses hours worked, and includes only households *always* on benefits. First, we explore whether our results are robust to the use of a different sample. Column (5) still restricts the sample to benefit recipients, but also includes households who may have changed benefit status over time. Second, we specify an alternative definition of benefit receipt. Although our definition of benefit receipt based on hours worked is likely to have very few false positives, we are likely to have a higher rate of false negatives. To consider whether this is important we use a wider set of characteristics available in both the Kantar data and in the Expenditure and Food Survey (EFS) to predict benefit receipt in the EFS (see Appendix B, and Table B2). We apply the estimated coefficients from the EFS to the Kantar data to create a predicted probability of benefit receipt. We define households as being on benefits when their predicted probability exceeds 0.7.

This approach also does a good job at capturing those who truly receive benefits: the EFS data shows that, among those defined as being on benefits (i.e. having a probability of benefit receipt that exceeds 0.7), 92% actually receive benefits (not shown here, but available upon request). Using this different sample of benefit recipients, we estimate the effect of the Healthy Start scheme on fruit and vegetable consumption. The results are presented in column (6) of Table C5. Finally, column (7) uses the full sample of households with children aged 0–8, specifying the probability of benefit receipt, as predicted from the EFS estimates, as weights in the analysis (Arellano and Meghir, 1992). This idea is similar to that used in propensity score matching in the policy evaluation literature: using the predicted probability of treatment (here: being on benefits) as weights in an ordinary least squares regression.

Our results are robust to these alternative specifications. We therefore believe that our results provide strong evidence that the effects of the reform operated through distorted households, not infra-marginal households, consistent with the underlying economic incentives in the policy.

## 6 Summary and discussion

Our analysis of the Healthy Start scheme makes a substantive contribution to the ongoing academic and policy debate about how to improve dietary choices. We identify that targeted benefits can be effective in increasing purchases of fruit and vegetables and, importantly, can improve the nutritional composition of food spending. Our regression result estimates show that the Healthy Start scheme has increased spending on fruit and vegetables by £2.43 per month (approx. $3.50), equivalent to a 15.5 per cent increase in spending compared to pre-reform levels. This is an Intention To Treat (ITT) estimate; with a take-up rate of 80%, this suggests that the treatment-on-the-treated (TOT) effect is £3.04, or 19.4%.

The estimated MPC out of vouchers is 0.144 (±0.285); in other words, each additional £1 of Healthy Start vouchers caused households to increase their spending on fresh fruit and vegetables by 14 pence. The effect we estimate is in line with the estimated effect of other targeted benefits, such as Food Stamps,^15^ although it might have been expected to be bigger given that most recipients of Food Stamps are infra-marginal (Hoynes and Schanzenbach, 2009, Whitmore, 2002) whilst the majority of Healthy Start voucher recipients are distorted. However, there are a number of reasons why this estimated magnitude is plausible. First, many households that are distorted spend only slightly less than the value of the vouchers (average monthly spending on fruit and vegetables by distorted eligible households in the pre-reform period was £15.53 compared to an average voucher value of £16.74). Second, as we mention above, our estimate is an ITT effect and thus a lower bound on the effect on recipient households. The advantage of focusing on eligible households rather than recipient households is that eligibility is solely determined by the (exogenous) age of children in the household, implying that our estimate is not upwardly biased by households selecting into the scheme. Third, there is likely to be some measurement error in our definition of eligibility: 80% of eligible households receive the vouchers, and we estimate that approximately 8% of our sample may not truly be eligible (i.e. receive means-tested benefits).

We have also shown that the scheme has been accompanied by an improvement in the nutritional composition of households' shopping baskets – with increases in the proportion of households meeting their recommended Reference Intakes, increases in purchases of a number of nutrients known to be important for child development (fibre, beta-carotene (vitamin A), vitamins C, D and E, potassium, iron and zinc) and no increase in purchases of nutrients that are generally classified as less healthy, and of which many households consume more than is recommended (fats and added sugars). This indicates that households did not substitute purchases of the targeted goods for purchases of close, healthy substitutes, nor did they increase spending on unhealthy foods, although there is some evidence that total calories might have increased.

There are a number of important caveats to our findings. First, we have a relatively small number of households, though the advantage of our data is that we can follow the same households over time. We also have detailed expenditure data, but do not directly measure household income and whether the household receives benefits; we derive the latter from the data on hours worked. Furthermore, we study whether the vouchers affect the *purchase* decisions of households. From a public health perspective, however, we would like to know what goes on inside the household. In particular, as the program specifically targets healthy eating for pregnant women and young children, it would be interesting to study the intra-household consumption of foods, exploring who consumes the additional fruit, vegetables and healthy nutrients. Unfortunately, there is no data that we are aware of that records both intra-household food allocation as well as information on benefit receipt and expenditure. Instead, the focus in this paper is on the purchasing decision. Indeed, vouchers are targeted at this decision, aiming to increase the amount of fruit and vegetables purchased by the household, which is of course an important precursor to observing increases in actual consumption.

Finally, we show that the vouchers increased spending among distorted households in line with standard economic incentive effects, not behavioural mechanisms. This is in line with other studies on the effects of information or promotional campaigns in relation to healthy eating (Bollinger et al., 2010; Capacci and Mazzochi, 2011 and Stables et al., 2002). Although small-scale experiments have suggested that ‘nudging’ might be effective in improving individuals' dietary choices (Downs et al., 2009; Wisdom et al., 2010; Wansink et al., 2011; Wansink and Just, 2011), our study suggests that more work is needed to understand if and why behavioural mechanisms affect dietary choices and how they can be exploited for the purposes of policy-making.

## Footnotes

1 Although the vouchers were for fruit, vegetables and milk, we focus our analysis on fruit and vegetables. This is in line with the focus of the policy on healthy eating. Also, as we show in Appendix A, milk tends to be consumed in fixed quantities by affected households; we might therefore expect the main response to be in fruit and vegetables.

2 In that sense, it is similar to WIC in the US, that introduced new food packages (including fruit and vegetable vouchers) in 2009. Evaluation of these changes suggest they improved dietary intakes (Whaley et al., 2012; Chiasson et al., 2013; Odoms-Young et al., 2014; Tester et al., 2016), with reductions in obesity among children (Chiasson et al., 2013). These findings confirm previous US studies, showing that targeted subsidies increase the consumption of fruits and vegetables (see e.g. Herman et al., 2006).

3 It was piloted from November 2005 in Devon and Cornwall. We drop households in the South West of England from our empirical analysis.

4 Households were also sent vouchers for vitamins for pregnant women and children. We do not consider this here, and our analyses on nutrients below exclude any such supplementation.

5 As a comparison, the average funding available per recipient through the US Special Supplemental Nutrition Program for Women, Infants, and Children is $700 per year, compared to approximately £240 ($400) for the average family receiving Healthy Start Vouchers.

6 The US Special Supplemental Nutrition Program for Women, Infants and Children is similar in that it provides vouchers that can only be spent on specific healthy foods.

7 This excludes household-month observations that have periods of non-recording longer than seven days, outliers in spending and quantity purchased, and households in the South-West; see below.

8 Purchases of fresh milk are included, but not formula milk. As discussed in the previous section, we assume that the reform was neutral with respect to purchases of formula.

9 Among the 80% of households in our sample that are asked to record loose fruit and vegetables, 99% do so at least once during our observation period; for 92% of all household-months, we observe loose items.

10 We here assume that any change in spending due to the scheme for the 20% of households is similar across loose and packaged items. If, on the other hand, these households increase their purchases of loose items, but do not change their purchases of packaged items, we would underestimate the effects of the scheme.

11 Women become eligible to receive vouchers from week 10 of pregnancy. However, the majority of midwife appointments (where women are made aware of the scheme) take place at 12 weeks gestation. We therefore consider eligibility for pregnant women to start at 12 weeks.

12 There is a strong correlation between grocery expenditure and income. We do not have good measures of household income in the same data as detailed expenditure on fruit and vegetables. Using the Living Costs and Food Survey we show that the correlation between the log of equivalized household income and grocery expenditure is 0.23 and is highly statistically significant. This relationship is robust to the inclusion of expenditure on food outside the home, suggesting that income and grocery expenditure have a strong relationship, and that low levels of grocery expenditure are driven largely by poverty, not by greater reliance on or substitution to food outside the home.

13 In the Kantar data, we directly measure calories, macronutrients (protein, fats, carbohydrates) and fibre. Using these data leads to similar results compared to using the information from DEFRA that is available for a larger number of nutrients.

14 One possibility, suggested by the behavioural literature, is that the vouchers might provide a signal to infra-marginal households that they were spending “enough” on fruit and vegetables. However, we think this is unlikely because a high profile “five a day” campaign was running at the same time and was incorporated into healthy eating messages from healthcare professionals (see e.g. Department of Health, Social Services and Public Safety, 2011). In addition, the vast majority of households (98% in our estimation sample) purchase fewer than the recommended five a day (see also Capacci and Mazzochi, 2011).

15 Hoynes and Shanzenbach estimate the MPC to be 0.167, for example. Looking at our expenditure elasticities, although these are lower than the estimates in Capacci and Mazzochi (2011) for fruit and vegetables, they are not out of line with estimated expenditure elasticities of foods in the literature (see e.g. Browning and Meghir, 1991; De Agostini, 2014).
