# Supplementary material for: Getting a healthy start: The effectiveness of targeted benefits for improving dietary choices
Source: J Health Econ. 2018 Mar;58:176–87. doi: 10.1016/j.jhealeco.2018.02.009 (PMC5887873; doi:10.1016/j.jhealeco.2018.02.009)
Supplement: Supplementary file 1 [file mmc1.pdf]

FOR ONLINE PUBLICATION

Appendix A: The Welfare Food scheme and the Healthy Start scheme

Welfare Food scheme

Concerns about access to nutritional foods during wartime rationing led to the introduction of the Welfare Food scheme (WFS) in the 1940s (Department of Health, 2002). The scheme provided free access to milk and vitamins for low-income pregnant women and low-income households with children under five. Households that received certain social security benefits were sent one token each week that could be exchanged for seven pints of liquid milk, or 900g of infant formula. These benefits included Income Support, Income-based Jobseeker's Allowance, or Child Tax Credit with a family income less than a year-specific threshold (e.g. £13,480 in the tax year 2004/05; see also Table 1). The scheme changed little over the first 60 years, but was substantially reformed in 2006, when it was replaced by the Healthy Start scheme.

Healthy Start scheme

The Healthy Start scheme replaced the Welfare Food scheme on 27 November 2006 after being piloted in Devon and Cornwall from November 2005. The scheme was explicitly intended to promote healthy lifestyles by changing diets. Households are sent vouchers that can be spent on plain fresh fruit and vegetables, cow's milk, or infant formula. Vouchers cannot be spent on fruit and vegetables that have any other ingredient added, such as sugar or seasonings as in potato salad, oven chips, battered onion rings, or seasoned vegetables. From 2011, they could additionally be spent on frozen fruit and vegetables; we do not consider that here. Furthermore, households were sent vouchers for vitamin tablets for pregnant women, and women with a baby under one year old, and they were sent vouchers for vitamin droplets for children from 6 months to four years old. We do not consider this here, and our analyses on nutrients exclude any such supplementation.

On introduction of the Healthy Start scheme in November 2006, the monetary value of a voucher was £2.80 (this gradually increased over time; see Table 1). Similar to the Welfare Food scheme, the receipt of certain benefits determines whether the family receives Healthy Start Vouchers, including Income Support, Income-based Jobseekers' Allowance, and Child Tax Credit with a family income less than a year-specific threshold.[1]

[1] From October 2008, the benefits also include Income-related Employment and Support Allowance. We do not consider that here, as our sample ends in November 2008.

Take-up and use of Healthy Start Vouchers is high. An estimated 79-80% of all eligible households receive the vouchers, of which 90% are used (Department of Health, 2009). Many retailers accept Healthy Start Vouchers, including supermarkets, corner shops, milkmen, chemists, market stalls and greengrocers. Households can find information about which retailers accept vouchers on the Healthy Start website, or by calling their helpline.

All applications to the Healthy Start scheme have to be countersigned by a health visitor or midwife, who is also expected to provide information and advice on breastfeeding and healthy eating. Eligible households are sent four vouchers per 4-week period per child (eight vouchers for children aged between 0 and 1). Vouchers are only valid for this four week period, after which they can no longer be used.

To date, there have not been any large-scale evaluations of the Healthy Start scheme, though there are three small (qualitative) studies. One focuses on the views and experiences of parents, professionals and small retailers (Lucas et al., 2013), whilst the others examine whether it affected food consumption. They find an increase in reported fruit and vegetable consumption (Hills et al., 2006) and intakes of energy, calcium, folate, iron and vitamin C (Ford et al., 2009), but sample sizes are small (n = 58 and 336 respectively).

Figure A1 illustrates the standard economic incentive effects of targeted benefits such as the Healthy Start scheme. For simplicity we consider the case without the Welfare Food scheme. The line A-A’ represents the initial budget constraint, where the household decides between spending on fresh fruit, vegetables and milk and spending on other goods. The introduction of the vouchers shifts the budget constraint outwards, but also introduces a kink, since the extra income can only be spent on the targeted goods, shown in line A-B-B’. The budget constraint for an equivalent value cash benefit without constraints on spending would be B’’-B’.

Consider first “distorted households”, represented by U_1, who would spend less than the value of the vouchers on fresh fruit and vegetables if they were given cash. Receipt of the vouchers leads these households to move out to ‘the kink’ (point B), increasing spending on the targeted good by more than if they were given a cash benefit (Southworth, 1945). For “infra-marginal households”, represented by U_2, however, the effect of the vouchers is the same as cash benefits. They use the voucher to cover existing spending and then re-allocate their (non-voucher) income among other goods. These consumers are also predicted to increase their spending on the targeted good, but the magnitude is in line with a standard income effect.

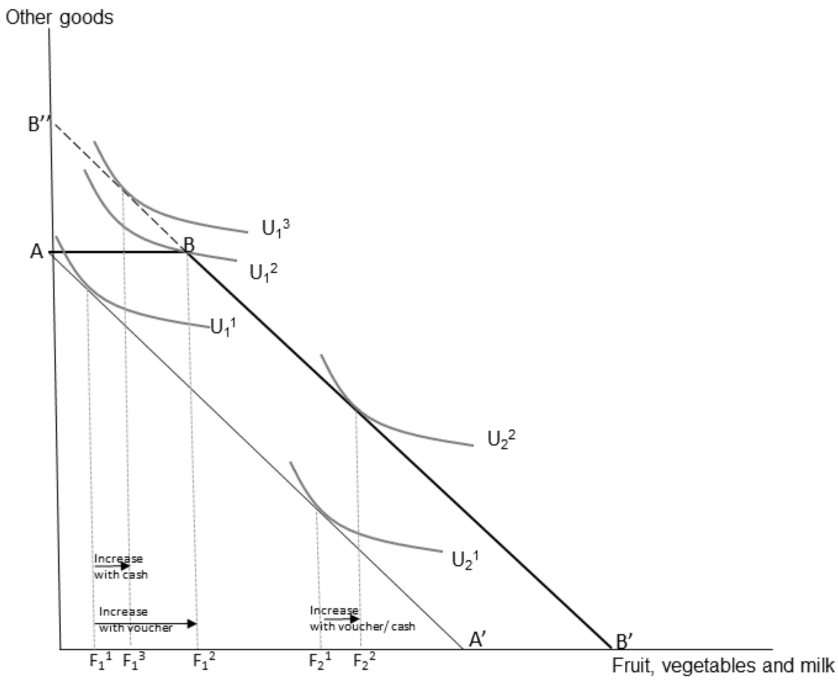

Figure A1: Effect of targeted benefits

Milk spending

The pre-existence of the Welfare Food scheme complicated the analysis slightly. We focus on fruit and vegetables. We argue that households consume milk in relatively fixed quantities, depending on household size. Our analyses in Table A1 below support this, showing the estimates of a simple difference-in-difference analysis, comparing milk spending of eligible and ineligible households before and after the introduction of the scheme (we discuss the estimation in more detail in Section 4). Column 2 presents the estimates of a triple differences analysis, additionally distinguishing between distorted and infra-marginal households. Column 3 and 4 show the same for the quantity of milk purchased (in litres). These analyses show no evidence that milk spending changed with the introduction of Healthy Start Vouchers.

Figure A2 below shows the distributions of monthly spending on milk and on fruit and vegetables, conditional on the number of adults, the number and ages of children, year, month and household fixed effects. This shows that the distribution of monthly milk expenditures is very concentrated, and much more concentrated than monthly spending on fruit and vegetables,

with no large differences between eligible, ineligible, distorted, or infra-marginal households. Analyses (not shown here, but available from the authors upon request) show that the standard deviation of milk spending is significantly smaller than the standard deviation of spending on fruit and vegetables.

We also confirm findings from the UK Committee on Medical Aspects of Food and Nutrition Policy (Department of Health, 2002), who state that the vast majority of households did not consume the amount of milk they could purchase with a token under the Welfare Food scheme. Our findings suggest 85% of households in our sample belong to this group, with little difference between eligible, ineligible, distorted and infra-marginal households. Finally, examining the effect of the introduction of the Healthy Start scheme on milk spending shows no significant effects, with all estimates close to zero.

The reform of the scheme

Estimating the effect of the introduction of Healthy Start Vouchers is complicated by the pre-existing Welfare Food scheme. We assume that consumers maximise their utility, which is a function of their spending on milk (x_1), fruit and vegetables (x_2) and other food (x_3): $U = U(x_1, x_2, x_3)$. This is subject to a budget constraint, given by

$$m + b = y = x_1 p_1 + x_2 p_2 + x_3 p_3,$$

where m denotes (other) income and b denotes income from benefits.

We assume milk is purchased in fixed quantities, depending on household size, the number and age of children: $x_1 = \bar{x}_1 = x_1(N_{ads},\ N_{kids}\ , Age_{kids})$. In other words, the quantity purchased does not depend on price or household income. This is consistent with the fact that the distribution of milk spending (conditional on household characteristics) is very concentrated, as shown above. If benefit income is paid in cash, households purchase a fixed quantity of milk and then allocate their remaining budget between fruit and vegetables and other food: $x_n^* = x_n(\ p_2,\ p_3, y - p_1 \bar{x}_1)$, with *n = 2, 3* and where the superscript * indicates the optimal spending.

Under the Welfare Food scheme, households receive welfare tokens of value b that can only be spent on milk. For the majority of households, the report by the UK Committee on Medical Aspects of Food and Nutrition Policy indicates that that $b < \bar{x}_1 p_1$ (Department of Health, 2002). In other words, households are “distorted”, they but are assumed not to locate at the kink because the amount of milk that can be purchased with the tokens is greater than the maximum they want to purchase. Because of the distortion, spending on fruit and vegetables and other

food for household receiving welfare tokens is given by $\hat{x}_n = \hat{x}_n(\, p_2,\, p_3, y - b) \leq x_n^*$, with $n = 2, 3$.

In November 2006, welfare tokens are replaced with Healthy Start Vouchers, which are of roughly equivalent monetary value. However, the new vouchers can be spent on milk, fruit and vegetables: $b = \bar{x}_1 p_1 + x_2 p_2$. The effect of introducing the vouchers is similar to the case with no welfare tokens (as shown in Figure A1), but the value of the extra benefit is lower: $b' = b - \bar{x}_1 p_1$.

We can distinguish between two groups. First, those who are infra-marginal under Healthy Start Vouchers: $b \leq \bar{x}_1 p_1 + x_2^* p_2$, and who therefore choose optimal spending $\bar{x}_1, x_2^*, x_3^*$. Following introduction of Healthy Start Vouchers, there is no change in spending on milk, while spending on fruit and vegetables and other food increase in line with b' (from $\hat{x}_n$ to x_n^*) due to an income effect.

Second, those who are distorted under Healthy Start Vouchers spend $\bar{x}_1 p_1$ on milk, $\check{x}_2 p_2 = (b - \bar{x}_1 p_1) \geq x_2^* p_2$ on fruit and vegetables and $\check{x}_3 = \check{x}_3(\bar{x}_1, \check{x}_2,\, p_3, m) < x_3^*$ on other food. This predicts that the increase in spending on fruit and vegetables is greater among distorted than among infra-marginal consumers (from $\hat{x}_2$ to $\check{x}_2$), whilst the increase in spending on other foods is less among distorted than infra-marginal consumers (from $\hat{x}_3$ to $\check{x}_3$).

Before the introduction of the reform, we observe $\bar{x}_1 p_1 + \hat{x}_2 p_2$. Using the cut-off b will therefore cause some distorted consumers to be included in the infra-marginal group, leading to an overestimate of any change in fruit and vegetable spending for the infra-marginal group.

Additional References Appendix:

Ford, Fiona, Theodora Mouratidou, Wademan Sarah, Fraser Robert. 2009. “Effect of the introduction of 'Healthy Start' on dietary behaviour during and after pregnancy: early results from the 'before and after' Sheffield study.” *British Journal of Nutrition*, 101(12):1828-36.

Hills D, Child C, Junge K, Wilkinson E, Sullivan F. 2006. *Healthy Start. Rapid evaluation of early impact on beneficiaries, health professionals, retailers and contractors.* London: Tavistock & Symbia.

Table A1: Effect on Milk Spending and Quantity

	(1)	(2)	(3)	(4)	(5)	(6)	(7)	(8)
Dependent variable:	milk expenditure		ln(milk expenditure)		milk quantity (in litres)		ln(milk quantity)	
Treatment effect	-0.031		0.021		-0.033		0.034	
	(0.402)		(0.058)		(0.740)		(0.059)	
Treatment effect (distorted)		0.094		0.054		0.274		0.069
		(0.454)		(0.060)		(0.835)		(0.062)
Treatment effect (infra-marginal)		-0.447		-0.088		-1.054		-0.085
		(0.639)		(0.093)		(1.178)		(0.095)
Household is eligible	0.454		0.002		0.701		-0.023	
	(0.412)		(0.059)		(0.755)		(0.060)	
Household is eligible (distorted)		0.348		-0.036		0.220		-0.073
		(0.505)		(0.073)		(0.951)		(0.076)
Household is eligible (infra-marginal)		0.750		0.109*		2.003**		0.115*
		(0.475)		(0.060)		(0.811)		(0.062)
Number of households	296	296	296	296	296	296	296	296
Number of household months	4976	4976	4976	4976	4976	4976	4976	4976

*Notes: Observation period runs from December 2004 - November 2008. All columns include household, month and year fixed effects, age and age squared of youngest and oldest child (in months), dummies for whether household includes: 2 adults, 3+ adults, 1 child, 2 children, 3 children, 4+ children, and a dummy indicating whether the household did not buy any milk that month. Eligible households are those with a child aged 0-3 or where the woman is ≥3 months pregnant. The post reform period equals 1 for the period December 2006 onwards. Robust standard errors in parentheses, clustered by household. * p<0.10, ** p<0.05, *** p<0.01.*

Figure A2: Conditional distributions of monthly expenditures on milk and on fruit and vegetables

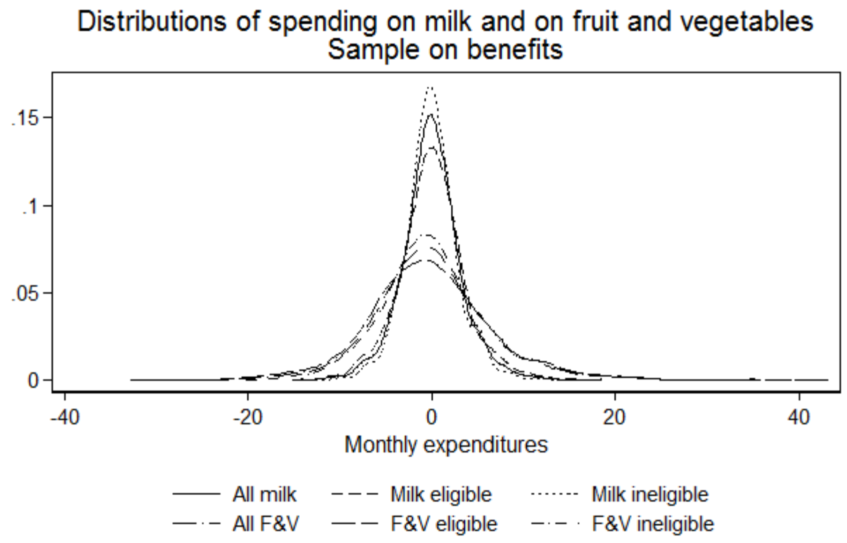

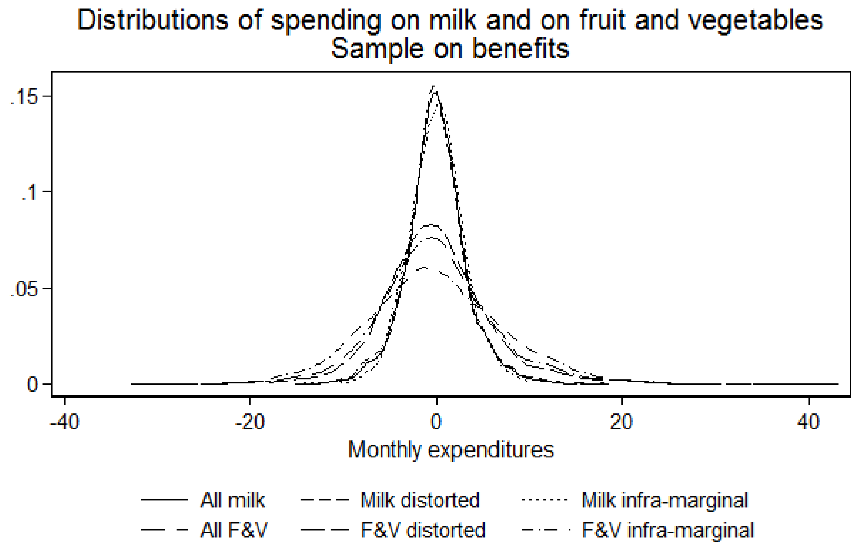

Note: Expenditure is conditional on the number of adults, the number and ages of children in the household, year, month and household fixed effects.

Appendix B: Identifying households on benefits in the Kantar data

We define a household as being "on benefits" if both the head and main shopper are not in work, unemployed, in education, or work less than 8 hours a week. To assess how well this simple rule does in predicting which households are on benefits, we look at data from the Expenditure and Food Survey (EFS; a repeated cross-sectional study of households in the UK), which contains both hours worked and actual benefits receipt. Using this definition, we find that 17.8% of all households with a child aged 0-8, or where the woman is pregnant in the EFS are on benefits, compared to 20.7% in a similar sample in the Kantar data. In addition, we compare our definition of being on benefits (based on hours worked) in the EFS to the share of these households that are truly on benefits. Table B1 shows that we do well in capturing households that are truly on benefits. The rows show the work status of the head of household, recoded to the categories observed in the Kantar data; the columns show the hours worked by the spouse (or that there is no spouse present in the household).

The shaded area indicates the sample used in our analysis: those we predict to be on benefits using hours worked. We show the number of households observed in each cell, the number who is in on benefits, and the probability that households in each cell are on benefits. For example, the upper left hand cell shows that of single parent households where the parent is not in work, 95% of households in the EFS are in receipt of benefits. Similarly, of households where the head works between 1-8 hours and there is no spouse 90.3% are on benefits. Overall, among those households in the EFS that we predict to be on benefits based on the hours worked, 91.7% actually received benefits.

We examine the robustness of our findings to an alternative definition of benefit receipt, where rather than using hours worked, we predict the probability of receiving benefits as a function of covariates in the EFS. Table B2 shows the marginal effects of a probit regression using the EFS for the period December 2004 to November 2008. This suggests that we predict benefit receipt well: a simple probit using the cross-sectional EFS provides a pseudo R^2 of 0.55.

Figure B1 compares the distribution of the predicted probability of being on benefits in the Kantar data (predicted using the estimates from the EFS) with that from the EFS data. This shows that they line up well: the majority of individuals are predicted not to be on benefits, with the largest densities for probabilities of benefit receipt less than 0.4. The density increases again for probabilities above around 0.7. In our robustness analysis, we use Pr(benefits)>0.7 as the cut-point to define a household as being on benefits, though the results are robust to using

different cut-points ranging from 0.6 to 0.9. Comparing this predicted benefit receipt (based on Pr(benefits)>0.7) to the actual benefit receipt in the EFS, we find that, among those we define as being on benefits, 92% receives benefits.

Figure B1: The densities of Pr(on benefits) from the EFS and Kantar data

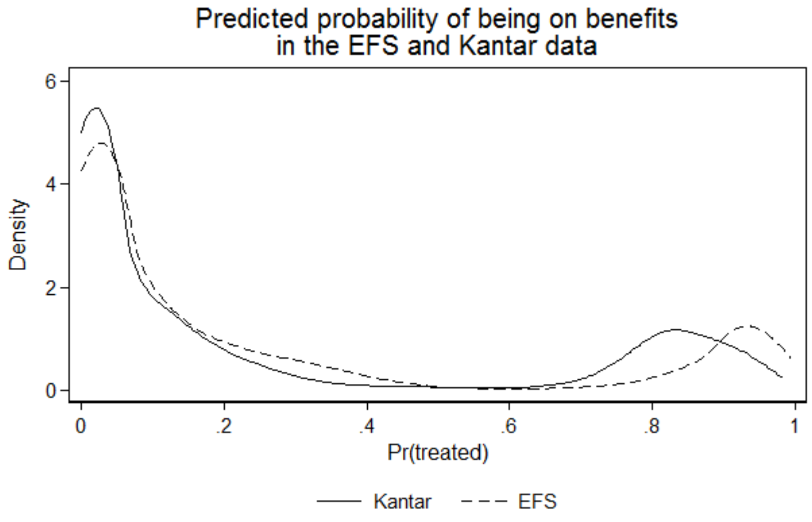

Table B1: Number and percent of households on benefits by demographic status (EFS)

Head:		Spouse: no spouse	not in work	1-8 hours	8-30 hours	30+ hours	Total
not in work	*N* in group	804	326	2	48	149	1,329
	N on benefits	764	284	1	25	33	1,107
	% on benefits	95.0%	87.1%	50.0%	52.1%	22.1%	83.3%
1-8 hours	*N* in group	31	6	0	2	7	46
	N on benefits	28	2	0	0	2	32
	% on benefits	90.3%	33.3%	-	0%	28.6%	69.6%
8-30 hours	*N* in group	313	109	5	43	181	651
	N on benefits	50	33	1	11	9	104
	% on benefits	16.0%	30.3%	20.0%	25.6%	5.0%	16.0%
30+ hours	*N* in group	250	1,242	106	1,540	1,160	4,298
	N on benefits	15	151	10	93	15	284
	% on benefits	6.0%	12.2%	9.4%	6.0%	1.3%	6.6%
Total	*N* in group	1,398	1,683	113	1,633	1,497	6,324
	N on benefits	857	470	12	129	59	1,527
	% on benefits	61.3%	27.3%	10.6%	7.9%	3.9%	24.1%

*Note: For each demographic group, the table provides the total number of households in that group, the number of households in that group who receive benefits, and the percent of households on benefits (i.e. (N on benefits/N in group)*100). The shaded area is the main sample definition used in the Kantar data. Calculations from the Expenditure and Food Survey.*

Table B2: Determinants of benefit receipt: marginal effects from a probit regression using the EFS

	Pr(on benefits)	
2 adults	-0.112*	(0.069)
3 adults	-0.079**	(0.025)
Pregnancy	-0.033*	(0.017)
No. aged 0	0.004	(0.019)
No. aged 1	0.011	(0.018)
No. aged 2	0.005	(0.019)
No. aged 3	0.004	(0.018)
No. aged 4	0.006	(0.019)
No. aged 5	0.007	(0.018)
No. aged 6	-0.000	(0.019)
No. aged 7	0.005	(0.019)
No. aged 8	0.002	(0.018)
No. aged 9	-0.022	(0.021)
No. aged 10	0.026	(0.020)
No. aged 11	0.018	(0.022)
No. aged 12	0.022	(0.027)
No. aged 13	0.006	(0.024)
No. aged 14	-0.041	(0.026)
No. aged 15	0.003	(0.031)
No. aged 16	0.044	(0.030)
No. aged 17	0.000	(0.037)
No. aged 18	0.062	(0.044)
Household size squared	-0.001	(0.002)
Married	-0.014	(0.054)
Widowed/divorced/separated	-0.026	(0.017)
Non-manual social class	0.201***	(0.053)
Manual social class	0.442***	(0.057)
Semi-skilled social class	0.364***	(0.070)
Unskilled social class	0.369***	(0.087)
Unemployed	0.551***	(0.082)
Head works less than 8 hours per week	-0.036	(0.041)
Head works between 8 and 30 hours per week	-0.123***	(0.011)
Head works 30 or more hours per week	-0.353***	(0.051)
Shopper works less than 8 hours per week	0.016	(0.031)
Shopper works between 8 and 30 hours per week	-0.097***	(0.011)
Shopper works 30 or more hours per week	-0.179***	(0.010)
Household owns 1 TV	0.029	(0.045)
Household owns 2 TVs	-0.002	(0.040)
Household owns 3 TVs	0.010	(0.042)
Household owns 4 TVs	0.008	(0.042)
Household owns 2 cars	0.015	(0.013)
Household owns 3 cars	0.043*	(0.025)
Household owns a PC	0.003	(0.016)
Household owns a freezer	0.006	(0.042)
Household owns a drier	0.010	(0.012)
Household has internet	-0.043***	(0.016)
Household owns a microwave	0.001	(0.025)
Household owns a dishwasher	-0.033***	(0.011)
Pseudo R^2	0.55	
Observations	6064	

*Notes: Regression uses data from the Expenditure and Food Survey (EFS) 2004-2008. Year and region dummies included. Standard errors in parentheses, * $p < 0.10$, ** $p < 0.05$, *** $p < 0.01$*

Appendix C: Additional tables

Table C1: Means and standard deviations of the Kantar data, by eligibility and distorted and infra-marginal households

	(1)		(2)		(3)	(4)
	Eligible		Ineligible			
	Mean	SD	Mean	SD	Diff	p-value
**Distorted**						
Total spending (£): fruit & vegetables	15.53	(9.9)	13.26	(10.4)	2.27	<0.01
Total quantity (kg): fruit & vegetables	15.78	(9.4)	12.92	(9.2)	2.86	<0.01
Proportion purchasing ≥5 portions per person/day	0.01	(0.1)	0.00	(0.1)	0.00	0.22
Total spending: all foods	180.03	(68.7)	168.64	(71.7)	11.39	<0.01
Household size	4.05	(0.9)	3.51	(1.0)	0.28	<0.01
≥3 months pregnant	0.08	(0.2)	-	-	-	N/A
No. of 0 year olds	0.21	(0.4)	-	-	-	N/A
No. of 1-3 year olds	0.92	(0.5)	-	-	-	N/A
No. of 4 year olds	0.15	(0.3)	0.29	(0.4)	-0.15	<0.01
No. of 5-18 year olds	1.06	(1.0)	1.64	(0.9)	-0.58	<0.01
No. of adults	1.94	(0.5)	1.70	(0.6)	0.24	<0.01
Number of household-month observations	1914		1728			
Number of households	149		141			
**Infra-marginal**						
Total spending: fruit & vegetables	27.94	(12.5)	23.56	(12.5)	4.38	<0.01
Total quantity (kg): fruit & vegetables	23.87	(9.6)	21.32	(10.3)	2.54	<0.01
Proportion purchasing ≥5 portions per person/day	0.03	(0.2)	0.07	(0.3)	-0.03	<0.01
Total spending: all foods	214.77	(62.0)	217.46	(67.7)	-2.68	0.45
Household size	3.65	(0.9)	3.37	(0.9)	0.28	<0.01
≥3 months pregnant	0.12	(0.3)	-	-	-	N/A
No. of 0 year olds	0.24	(0.4)	-	-	-	N/A
No. of 1-3 year olds	0.83	(0.5)	-	-	-	N/A
No. of 4 year olds	0.09	(0.3)	0.23	(0.4)	-0.14	<0.01
No. of 5-18 year olds	0.58	(0.7)	1.37	(0.9)	-0.80	<0.01
No. of adults	1.97	(0.6)	1.92	(0.6)	0.05	0.48
Number of household-month observations	596		738			
Number of households	58		67			

Note: Eligible households are those ≥3 months pregnant, or with a child aged 0-3; Ineligible households are those with children aged 4-8, or not yet pregnant. Households who (at any point prior to the reform) spent less than £12.86 per child on milk, fruit and vegetables are defined as distorted; those never spending less than £12.86 per child are defined as infra-marginal. The number of eligible and ineligible distorted and infra-marginal households exceeds the total number of households, as eligibility is time-varying, so households can be eligible in one month and ineligible in another. A portion of fruit and vegetables is defined as 80g. Other covariates, including marital status, social class, and the region where the household lives are not significantly different across distorted and infra-marginal consumers (not shown here, but available upon request).

Table C2: The effect of Healthy Start Vouchers: Common trend assumption

	(1)	(2)
	Placebo: Dec04 – Nov06	
Dependent variable:	F&V expenditure	F&V quantity
Treatment effect (per £), β_3 in eqn (2)	0.011	-0.021
	(0.048)	(0.044)
Value of voucher (in £), β_2 in eqn (2)	-0.096*	-0.072
	(0.049)	(0.045)
Post reform, β_1	-0.924	-0.847
	(1.102)	(1.059)
No. of households	288	288
No. of household-months	2593	2593

*Notes: The introduction of the scheme is defined as November 2005. All columns include household, month and year fixed effects, age and age squared of youngest and oldest child (in months), dummies for whether household includes: 2 adults, 3+ adults, 1 child, 2 children, 3 children, 4+ children, and a dummy indicating whether the household did not buy any fruit and vegetables that month. Eligible households are those with a child aged 0-3 or where the woman is ≥3 months pregnant. The post period refers to December 2005 onwards. Robust standard errors in parentheses, clustered by household. * p<0.10, ** p<0.05, *** p<0.01.*

Table C3: The effect of Healthy Start Vouchers: treatment effect heterogeneity

	(1)	(2)	(3)	(4)
Dependent variable:	fruit and vegetable expenditure		fruit and vegetable quantity (kg)	
Treatment effect (distorted households), β_3 in eqn (3)	2.831***		2.296***	
	(0.642)		(0.661)	
Treatment effect (infra-marginal households), β_5 in eqn (3)	1.074		0.100	
	(1.380)		(1.164)	
Household is eligible (distorted), β_2 in eqn (3)	-3.389***		-2.551***	
	(0.888)		(0.798)	
Household is eligible (infra-marginal), β_4 in eqn (3)	-0.687		0.381	
	(1.299)		(0.956)	
Treatment effect (per £, distorted), β_3 in eqn (4)		0.087***		0.086***
		(0.031)		(0.029)
Treatment effect (per £, infra-marginal), β_5 in eqn (4)		0.065		0.047
		(0.050)		(0.044)
Total grocery spending (distorted, in £), θ^D in eqn (4)		0.060***		0.053***
		(0.003)		(0.003)
Total grocery spending (infra-marginal, in £), θ^{IM} in eqn (4)		0.067***		0.049***
		(0.008)		(0.008)
Number of households	296	296	296	296
Number of household-months	4976	4976	4976	4976

*Notes: Column (1) and (3) show esimates of the coefficients from equation (3), columns (2) and (4) show estimates of the coefficients from equation (4), observation period runs from December 2004 - November 2008. All columns include household, month and year fixed effects, age and age squared of youngest and oldest child (in months), dummies for whether household includes: 2 adults, 3+ adults, 1 child, 2 children, 3 children, 4+ children, and a dummy indicating whether the household did not buy any fruit and vegetables that month. Eligible households are those with a child aged 0-3 or where the woman is ≥3 months pregnant. The post reform period refers to December 2006 onwards. "D" indicates distorted households, "IM" indicates infra-marginal households. Total grocery spending is spending on food and fast moving consumer goods. Robust standard errors in parentheses, clustered by household. * p<0.10, ** p<0.05, *** p<0.01.*

Table C4: Spillover effects

	(1)	(2)	(3)	(4)	(5)	(6)	(7)
Dependent variable: Expenditure (in £) on:	Fruit juice	Frozen F&V	Prepared sweet foods	Prepared savoury foods	Crisps	Non-diet drinks	Diet drinks
Treatment effect (per £), β_3 in eqn (2)	-0.028*	-0.006	0.003	-0.001	0.014	0.025	0.003
	(0.016)	(0.015)	(0.049)	(0.047)	(0.015)	(0.019)	(0.018)
Value of voucher (in £), β_2 in eqn (2)	-0.016	-0.005	-0.009	0.030	-0.026	-0.016	0.008
	(0.017)	(0.012)	(0.043)	(0.048)	(0.016)	(0.021)	(0.018)
Post reform, β_1	0.073	0.450	0.704	0.090	-0.293	0.279	-0.636
	(0.512)	(0.345)	(1.991)	(1.545)	(0.480)	(0.614)	(0.593)
Mean monthly pre-scheme spending (in £)	3.14	5.03	27.98	31.31	5.80	6.25	7.21
No. of households	296	296	296	296	296	296	296
No. of household-months	4976	4976	4976	4976	4976	4976	4976

	(8)	(9)	(10)	(11)	(12)	(13)	(14)
Dependent variable: Expenditure (in £) on:	Grains	Dairy	Cheese	Red Meats	Poultry and Fish	Alcohol	Non-food
Treatment effect (per £), β_3 in eqn (2)	-0.027	-0.016	0.000	-0.018	0.013	0.012	-0.042
	(0.027)	(0.027)	(0.021)	(0.041)	(0.030)	(0.060)	(0.060)
Value of voucher (in £), β_2 in eqn (2)	-0.001	-0.011	0.001	0.000	-0.014	0.030	0.150**
	(0.027)	(0.021)	(0.020)	(0.038)	(0.028)	(0.055)	(0.061)
Post reform, β_1	-0.220	-0.016	-0.521	-0.789	0.757	-2.489	4.006*
	(0.715)	(0.522)	(0.576)	(1.146)	(0.987)	(2.642)	(2.157)
Mean monthly pre-scheme spending (in £)	17.40	7.28	9.44	20.49	10.44	11.79	39.26
No. of households	296	296	296	296	296	296	296
No. of household-months	4976	4976	4976	4976	4976	4976	4976

*Notes: All columns include household, month and year fixed effects, age and age squared of youngest and oldest child (in months), dummies for whether household includes: 2 adults, 3+ adults, 1 child, 2 children, 3 children, 4+ children, and a dummy indicating whether the household did not buy any fruit and vegetables that month. Eligible households are those with a child aged 0-3 or where the woman is ≥3 months pregnant. The post reform period refers to December 2006 onwards. Robust standard errors in parentheses, clustered by household. * p<0.10, ** p<0.05, *** p<0.01.*

Table C5: The effect of Healthy Start Vouchers: Functional form

Dependent variable:	(1)<br>Fruit and vegetable expenditures	(2)<br>ln(Fruit and vegetable expenditures)	(3)<br>Fruit and vegetable quantity	(4)<br>ln(Fruit and vegetable quantity)
Treatment effect, β_3 in eqn (1)	2.425***	0.155***	1.789***	0.130**
	(0.643)	(0.047)	(0.647)	(0.051)
Household is eligible, β_2 in eqn (1)	-2.659***	-0.084	-1.762**	-0.069
	(0.757)	(0.056)	(0.698)	(0.052)
Post reform, β_1	-0.671	0.015	-0.460	0.025
	(0.820)	(0.070)	(0.898)	(0.074)
Number of households	296	296	296	296
Number of observations	4976	4976	4976	4976

*Notes: Columns (1) and (3) are from Table 3 in the paper. Columns (2) and (4) repeat these specifications in logs. Sample includes 4976 observations on 296 households between December 2004 - November 2008. All columns include household, month and year fixed effects, age and age squared of youngest and oldest child (in months), dummies for whether household includes: 2 adults, 3+ adults, 1 child, 2 children, 3 children, 4+ children, and a dummy indicating whether the household did not buy any fruit and vegetables that month. Eligible households are those with a child aged 0-3 or where the woman is ≥3 months pregnant. The post-reform period refers to December 2006 onwards. Robust standard errors in parentheses, clustered by household. * p<0.10, ** p<0.05, *** p<0.01.*

Table C6: The effect of Healthy Start Vouchers: Robustness

	(1)	(2)	(3)	(4)	(5)	(6)	(7)
	Original specification	Drop hhs not recording loose F&V	Value of voucher minus milk spending	Expenditure shares	Include those on benefits at any point	Benefits defined as Pr(ben)>0.7	Benefit probability used as weights
Dependent variable:	F&V spending	F&V spending	F&V spending	Share of spending on F&V (x100)	F&V spending	F&V spending	F&V spending
Treatment effect (per £)	0.082***	0.078**	0.085**	0.048***	0.080***	0.067**	0.082***
	(0.029)	(0.038)	(0.036)	(0.013)	(0.027)	(0.032)	(0.023)
Post reform	-0.581	-0.740	-0.186	-0.054	-1.006	-0.852	-0.511
	(0.731)	(0.898)	(0.707)	(0.326)	(0.682)	(0.763)	(0.582)
Value of voucher (in £)	-0.101***	-0.102***	-0.119***	-0.034**	-0.088***	-0.079**	-0.084***
	(0.034)	(0.039)	(0.035)	(0.015)	(0.031)	(0.038)	(0.025)
Total grocery spending (in £)	0.062***	0.065***	0.061***	-0.004***	0.063***	0.060***	0.062***
	(0.003)	(0.004)	(0.003)	(0.001)	(0.003)	(0.003)	(0.002)
No. of households	296	219	296	296	457	254	1584
No. of household-months	4976	3999	4976	4976	6212	4266	27037

*Notes: F&V denotes fruit and vegetables. All columns include household, month and year fixed effects, age and age squared of youngest and oldest child (in months), dummies for 2 adults, 3+ adults, 1 child, 2 children, 3 children, 4+ children, and a dummy indicating whether the household did not buy any fruit and vegetables that month. The post reform period refers to December 2006 onwards. Total grocery spending is spending on foods and fast moving consumer goods (goods purchased in supermarkets). Column 1 repeats the analysis from Table 3 for comparison. Column 2 drops households that do not record purchases of loose fruit and vegetables. Column 3 defines the value of the voucher as the value minus spending on milk. Column 4 uses the share of spending on fruit and vegetables (x100) as the dependent variable. Column 5 also includes households whose benefit status changes over time. Column 6 uses households with a predicted probability of receiving benefits (using the EFS) over 0.7. Column 7 uses these predicted probabilities as weights. * p<0.10, ** p<0.05, *** p<0.01.*
